# Q fever seroprevalence in Australia suggests one in twenty people have been exposed

**DOI:** 10.1017/S0950268820000084

**Published:** 2020-02-05

**Authors:** H. F. Gidding, C. Q. Peng, S. Graves, P. D. Massey, C. Nguyen, J. Stenos, H. E. Quinn, P. B. McIntyre, D. N. Durrheim, N. Wood

**Affiliations:** 1 Women's and Babies Research, Kolling Institute, Northern Sydney Local Health District, St Leonards NSW 2065, Australia; 2 The University of Sydney Northern Clinical School, Sydney, Australia; 3 National Centre for Immunisation Research and Surveillance, Westmead, New South Wales, Australia; 4 School of Public Health and Community Medicine, UNSW Medicine, UNSW, Sydney, Australia; 5 Australian Rickettsial Reference Laboratory, WHO Collaborating Centre for Reference & Research on Rickettsioses, University Hospital Geelong, Victoria, Australia; 6 NSW Health Pathology, Nepean Hospital, Penrith, NSW, Australia; 7 Hunter New England Local Health District, NSW Ministry of Health, New South Wales, Australia; 8 School of Health, University of New England, New South Wales, Australia; 9 University of Newcastle, Wallsend, New South Wales, Australia; 10 Discipline of Child and Adolescent Health. Faculty of Medicine and Health, University of Sydney, New South Wales, Australia

**Keywords:** Australia, *Coxiella burnetii*, Q fever, seroprevalence

## Abstract

Q fever (caused by *Coxiella burnetii*) is thought to have an almost world-wide distribution, but few countries have conducted national serosurveys. We measured Q fever seroprevalence using residual sera from diagnostic laboratories across Australia. Individuals aged 1–79 years in 2012–2013 were sampled to be proportional to the population distribution by region, distance from metropolitan areas and gender. A 1/50 serum dilution was tested for the Phase II IgG antibody against *C. burnetii* by indirect immunofluorescence. We calculated crude seroprevalence estimates by age group and gender, as well as age standardised national and metropolitan/non-metropolitan seroprevalence estimates. Of 2785 sera, 99 tested positive. Age standardised seroprevalence was 5.6% (95% confidence interval (CI 4.5%–6.8%), and similar in metropolitan (5.5%; 95% CI 4.1%–6.9%) and non-metropolitan regions (6.0%; 95%CI 4.0%–8.0%). More males were seropositive (6.9%; 95% CI 5.2%–8.6%) than females (4.2%; 95% CI 2.9%–5.5%) with peak seroprevalence at 50–59 years (9.2%; 95% CI 5.2%–13.3%). Q fever seroprevalence for Australia was higher than expected (especially in metropolitan regions) and higher than estimates from the Netherlands (2.4%; pre-outbreak) and US (3.1%), but lower than for Northern Ireland (12.8%). Robust country-specific seroprevalence estimates, with detailed exposure data, are required to better understand who is at risk and the need for preventive measures.

## Introduction

Q fever is a zoonotic disease caused by the highly infectious bacterium *Coxiella burnetii*, which has an almost world-wide distribution. *C. burnetii* infects both wild and domestic animals and their ticks, and humans are exposed by inhalation of infected droplets or dust. Most (20%–80%) infections are asymptomatic but when illness does occur the symptoms are non-specific; ranging from a self-limiting influenza-like illness, sometimes with raised liver enzymes, to more severe symptoms of pneumonia, hepatitis and endocarditis [1].

In Australia, Q fever has been a notifiable disease in humans since 1977 [2], and in the past 5 years (2013–2018) there have been on average 517 cases reported annually (notification rate 2.1/100 000) [3]. However, there is a consensus that Q fever notifications underestimate infection rates, due to the asymptomatic nature of many acute infections, as well as underestimating disease rates, because the signs and symptoms are non-specific and diagnosis relies on clinicians suspecting Q fever, and ordering appropriate tests. A recent study among Australian blood donors estimated that 29%–39% of people with symptomatic Q fever in the past had not been diagnosed with the disease [4].

Serosurveys (*C. burnetii* antibody prevalence) provide a way of measuring past exposure that is unbiased by diagnostic testing patterns or symptomology. Several countries including Australia have conducted Q fever serosurveys in specific geographic regions [4–7] and high risk populations [8 9]. However, there have only been a handful of national serosurveys [10–15], especially across all ages [14] or in highly urbanised countries [10 12 14]. The aim of this study was to measure *C. burnetii* seroprevalence in a representative sample of the Australian population. Such data are of particular relevance in Australia, the only country where a Q fever vaccine (QVax^®^) is licensed for human use, and recommended for certain high-risk populations (mostly occupation-based exposure to animals) [16].

## Methods

### Population and study design

The serosurvey utilised a bank of 12 411 sera and plasma specimens collected opportunistically from 32 diagnostic testing laboratories around Australia in 2012 and 2013. Information available on each specimen included gender, age or date of birth, residential postcode and date of collection: a unique identifier was used to ensure that only one sample from any subject was tested. Subjects who were immunocompromised, had received multiple transfusions in the past 3 months, or were known to be infected with human immunodeficiency virus were excluded from the collection.

### Sample size calculations

Sample sizes were calculated based on the expected proportions of individuals seropositive for the *C. burnetii* phase II IgG antibody at a national level in each of the following age groups: 1 to 9, 10 to 14, 15 to 19, 20 to 24, 25 to 29, 30 to 39, 40 to 49, 50 to 59 and 60–79 years. A sample size of 200 specimens per age group was estimated to achieve a 95% confidence interval (CI) of ⩽±3% for each age group with a prevalence of up to 5% and ⩽±4% for a prevalence of up to 9%. A total sample of 1800 would produce a CI of ⩽±1.1% for an estimate of Q fever seroprevalence for Australia in the expected range of 1%–5% and to detect a minimum of 3.6% difference between seroprevalence in non-metropolitan and metropolitan regions (with 80% power and a 5% significance level; assuming seroprevalence was no more than 5% in metropolitan regions and knowing that approximately two-thirds of the Australian population lived in metropolitan regions) [17]. Within each age group, the sample was stratified to be proportional to the 2012 Australian population distribution by state and territory [18], and Australian Statistical Geography Standard remoteness classification [17], and equal numbers of males and females were sampled.

### Laboratory methods

Q fever serology was performed using an indirect immunofluorescence (IF) test by the Australian Rickettsial Reference Laboratory according to methods previously described [5]. Briefly, phase II antigen from *C. burnetii* (clone 4 of 9-mile strain) grown in the VERO cell line was affixed to a glass slide and incubated with a 1/50 dilution of sera. Fluorescein isothiocyanate-conjugated goat anti-human immunoglobulin was then used to detect the Phase II IgG antibodies against *C. burnetii*, and a positive (fluorescence) defined as a titre of ⩾50.

### Statistical analysis

Crude estimates for the proportion of specimens positive for the Phase II IgG antibody against *C. burnetii* were calculated separately by age group and gender. Crude estimates by remoteness and state/territory of residence are also provided in Supplementary Tables S1 and S2. Remoteness was based on mapping postcode of residence to the Accessibility/Remoteness Index of Australia (ARIA) [17]. ARIA includes measures of each locality's access to services based on road distance measurements from over 12 000 populated localities to the nearest Service Centres. ARIA is usually classified into five categories: major cities, inner regional, outer regional, remote and very remote. To obtain age standardised national and metropolitan (major cities)/non-metropolitan (very remote, remote, inner and outer regional categories of remoteness combined) seroprevalence estimates, the age group specific estimates were weighted by the age distribution of 1–79 year olds in the 2012 Australian population [18]. The normal approximation to the binomial method and the method by Lohr *et al*. [19] were used to estimate 95% CIs for crude and standardised estimates, respectively.

### Ethical approval

Approval was obtained from the Blood Service Human Research Ethics committee (2014#09) and the Sydney Children's Hospitals Network Human Research Ethics Committee (LNR/14/SCHN/409).

## Results

### Representativeness

The proportion of tested sera was consistent with the 2012 Australian population in terms of geographic distribution, except that there was an under representation from one small state (Tasmania) and slightly higher proportions from remote, and fewer from inner regional, regions (Tables 1 and 2). There were similar numbers of males and females tested in each age group (Table 3).

**Table 1.** Distribution of serosurvey samples and Australian population [18] by state and territory

State/territory	Serosurvey		% Australian population by state/territory
	*N* tested	% tested by state/territory (95% CI)	
Australian Capital Territory	31	1.7 (1.1–2.3)	1.7
New South Wales	576	32.3 (30.1–34.4)	32.1
Northern Territory	19	1.1 (0.6–1.5)	1.0
Queensland	364	20.4 (18.5–22.3)	20.1
South Australia	139	7.8 (6.5–9.0)	7.2
Tasmania	10	0.6 (0.2–0.9)	2.3
Victoria	441	24.7 (22.7–26.7)	24.9
Western Australia	205	11.5 (10.0–13.0)	10.6
Total	1785	100.0	100.0

**Table 2.** Distribution of serosurvey samples and population by remoteness^a^

Remoteness category	Serosurvey		% Australian population by remoteness
	*N* tested	% tested (95% CI) by remoteness	
Very remote	19	1.1 (0.6–1.5)	0.9
Remote	32	1.8 (1.2–2.4)	1.4
Outer regional	204	11.4 (10.0–12.9)	9.0
Inner regional	295	16.5 (14.8–18.3)	18.3
Major cities	1233	69.1 (66.9–71.2)	70.4
Total	1785	100	100

a Remoteness Areas of Australia based on the mapping postcode of residence to the ARIA [17].

**Table 3.** Numbers tested by gender and Q fever seroprevalence by the age group, 2012–13

Age group	*N* tested			*N* positive (male + female)	% positive (95% CI) (male + female)
	Male	Female	Total		
1–9	106	100	206	2	1.0 (0–2.6)
10–14	96	102	198	5	2.5 (0.3–4.7)
15–19	98	98	196	13	6.6 (3.1–10.1)
20–24	94	96	190	14	7.4 (3.7–11.1)
25–29	100	99	199	11	5.5 (2.4–8.7)
30–39	95	102	197	8	4.1 (1.3–6.8)
40–49	97	101	198	15	7.6 (3.9–11.3)
50–59	96	99	195	18	9.2 (5.2–13.3)
60–79	101	105	206	13	6.3 (3.0–9.6)
Total	883	902	1785	99	5.6 (4.5–6.8)^a^

a Population prevalence for 1–79 years weighted to be representative of the 2012 Australian population by the age group [20].

### Seroprevalence

There were 99 samples positive for the phase II IgG antibody against *C. burnetii.* This yielded an overall age standardised seroprevalence of 5.6% (95% CI 4.5%–6.8%), which did not differ significantly between metropolitan (5.5%; 95% CI 4.1%–6.9%) and non-metropolitan regions (6.0%; 95% CI 4.0%–8.0%). Seroprevalence was highest in the 40–49 (7.6%; 95% CI 3.9%–11.3%) and 50–59 (9.2%; 95% CI 5.2%–13.3%) year age groups, with a secondary peak in 20–24 year olds (7.4%; 95% CI 3.7%–11.1%; Table 3). There was a marked increase in seroprevalence between the ages of 10–14 and 15–19 years (2.5% *v* 6.6%; *P* = 0.051). More males were seropositive (6.9%; 95% CI 5.2%–8.6%) than females (4.2%; 95% CI 2.9%–5.5%). The point estimates of crude seroprevalence by state/territory and remoteness vary considerably, however the CIs are wide (Tables S1 and S2).

## Discussion

This is the first national Q fever serosurvey in Australia. Standardised seroprevalence estimates of above 5% were higher than expected and did not differ appreciably between rural and metropolitan regions. If extrapolated to the total estimated Australian population of 23.4 million [21], our data indicate exposure of an estimated 1.3 million people to *C. burnetti*. While it is not possible to obtain an accurate estimate of the burden of Q fever using these data alone, based on published estimates of clinical illness among exposed adults (~40%) and children (~12.5%) [22–24], this roughly translates to ~ 500 000 cases of acute Q fever-related illness. Given there were fewer than 12 000 Q fever notifications between 1991 and 2013 [3], our study suggests that Q fever is an under-recognised and important public health problem in Australia.

Few countries have conducted national serosurveys and seroprevalence estimates vary considerably (Table 4). Our estimate of 5.6% is higher, but of a similar magnitude, to that reported for the US (3.1%; *n* = 4437) [10], and in the Netherlands prior to a large outbreak (2.4%; *n* = 5654) [14], but lower than a study of comparable size in Northern Ireland (12.8%; *n* = 2394) [12]. Smaller national serosurveys in Cyprus, Bhutan and American Samoa are even more varied with reported seroprevalence estimates of 52.7% (*n* = 583), 6.9% (*n* = 864) and 0% (*n* = 197), respectively [11 13 15]. This magnitude of variation highlights the need for country specific serosurveys, although variations in population sampling (geographical and age-related), and laboratory test methods may account for some of the observed differences.

**Table 4.** Published national serosurveys examining seroprevalence of the Phase II IgG antibody against *C. burnetii*

Country	Year	Sampling method	Sample size	Age range (years)	Test method; cut off	Standardised % seropositive (95% CI)
Australia	2012–13	Opportunistic	2785	1–79	IF (in house); 1/50	5.6 (4.5–6.8)
Netherlands [14]	2006–07	Population-based^a^	5654	0–79	ELISA (Virion/Serion); ⩾20 U/ml IF (Focus Diagnostics); 1/32^b^	2.4 (NA)^c^
USA [10]	2003–04	Population-based	4437	⩾20	ELISA (Pan Bio Inc); NA^d^ IF (in house); 1/16	3.1 (2.1–4.3)^c^
Northern Ireland [12]	1987–88	Population-based^a^	2394	12–64	ELISA (Vircell); NA	12.8 (NA)^e^
Bhutan [15]	2015	Population-based	864	⩾13	IF (in house); 1/50	6.9 (NA)^e^
Cyprus [13]	NA (pre 2006)	Population-based	583	All ages	IF (bioMérieux); 1/60	52.7 (NA)^e^
American Samoa [11]	2010	Population-based^a^	197	⩾17	IF (in house); 1/50	0 (0–1.9)

CI, confidence interval; IF, indirect immunofluorescence test; NA, not provided.

a Utilised sera collected for another purpose.

b Performed on all ELISA positive/equivocal and random sample of negative sera.

c Adjusted using results of the IF test as the gold standard.

d Performed on all ELISA positive/equivocal sera.

e Crude estimate only provided.

In our serosurvey, seroprevalence increased noticeably between the ages of 10–14 and 15–19 years and peaked in 50–59 year olds. This pattern is in keeping with findings from previous regional Australian serosurveys [4 7], and with notifications of clinical cases of Q fever [25]. However, it is in contrast to linear increases in age specific seroprevalence reported in the two largest national serosurveys to date in the US and Netherlands [10 14]. Reasons for the different age-specific patterns in Australia are unclear and further studies are needed to understand why notifications and seroprevalence peak in middle-aged adults. Currently, QVax^®^ is only licensed in Australia for ages 15 years and older [16], but the rapid rise in seroprevalence between ages 10–14 and 15–19 years suggests that a number of children aged 15 years (and older) are being infected with *C. burnetii*. However, before any vaccination of children, further studies are needed to license the vaccine for this age group and more accurately estimate the burden preventable by vaccination, given children are less likely to be symptomatic or suffer severe disease compared with adults [22–24 26].

Australian studies comparing seroprevalence in rural and metropolitan regions provide conflicting results. The current study found a similar seroprevalence in rural and metropolitan regions (6.0% and 5.5%, respectively), in keeping with another opportunistic serosurvey in Queensland (5.3% and 5.0%, respectively) [7] but in contrast to a serosurvey among blood donors in the Australian states of New South Wales (NSW) and Queensland [4]. Using the same laboratory method that we used in our national study, the blood donor serosurvey reported seroprevalence estimates that were higher in rural *vs.* metropolitan donors in both Queensland (4.9% *vs.* 1.6%) and NSW (3.7% *vs.* 2.8%). The lower seroprevalence estimates among blood donors may be because donors are generally healthier than patients providing pathology samples for opportunistic serosurveys, and thus less likely to have been recently exposed to *C. burnetii*. However, this does not explain the regional differences which are probably due to variations in the areas sampled (select NSW and Queensland rural regions known to have high Q fever notification rates *versus* a national sample).

Most previous serosurveys report a higher seropositivity in males than females [4 5 7 10 12 14]. Our male: female ratio of 1.6:1 is similar to the US national serosurvey [10] and regional serosurveys conducted in Australia across a broad age range [4 7]. It is likely that higher seroprevalence in males is related to greater occupational contact with animals, consistent with equivalent seropositivity by gender reported among children in a regional Australian serosurvey [6]. In contrast, among notified cases of Q fever the male: female ratio is 4:1 [25] suggesting either a greater susceptibility to illness in males [27] and/or a diagnostic bias towards occupation-based risk groups.

Key strengths of the current study are its size, inclusion of all ages and geographic representativeness, enabling the calculation of a robust age standardised estimate of national Q fever seroprevalence. To our knowledge, only the Dutch have conducted such a large survey across all ages [14]. However, there are some limitations with our study. First, being an opportunistic collection there is the potential for selection bias. We tried to minimise any biases by sampling sera submitted for a wide range of routine pathology tests from large public and private laboratories located throughout Australia that serviced mostly ambulatory patients. A study in one state of Australia (Victoria) reported no significant differences in seroprevalence for a range of vaccine preventable diseases between a prospectively conducted random cluster survey and our first opportunistic serosurvey [28], suggesting any selection biases are minimal. Second, because the sera were opportunistically collected there was no information on risk factors for exposure or vaccination status. However, vaccine-induced antibodies are unlikely to have contributed significantly to the seroprevalence as groups recommended for vaccination make up a very small percentage of the population, and uptake, even among at-risk groups, is low (10% in a recent Australian survey) [4]. Third, the serosurvey was not powered to provide precise seroprevalence estimates for specific age groups or geographic regions (i.e. by state/territory or remoteness). This could mean that some of the differences in point estimates between smaller geographic regions are due to chance variations in sampling. Fourth, comparisons with other studies are difficult due to differences in laboratory testing methods and cut-offs (Table 4). Finally, not everyone exposed to *C. burnetii* would have antibodies detected; only 39% of blood donors reporting a past Q fever diagnosis tested seropositive [4], suggesting our results are a minimum estimate of past exposure.

In conclusion, we provide a robust estimate of Q fever seroprevalence in Australia that suggests a considerable burden of past exposure (and associated morbidity) which is not limited to rural areas. Furthermore, levels of seroprevalence among adolescents confirm that Q fever is an ongoing public health issue in Australia. Robust country-specific seroprevalence estimates, with detailed exposure data, are needed to better understand who is at risk, what drives risk and the need for preventive measures.

## Supplementary material

For supplementary material accompanying this paper visit https://doi.org/10.1017/S0950268820000084. click here to view supplementary material
