# Supplementary material for: Q fever seroprevalence in Australia suggests one in twenty people have been exposed
Source: Epidemiol Infect. 2020 Feb 5;148:e18. doi: 10.1017/S0950268820000084 (PMC7019564; doi:10.1017/S0950268820000084)
Supplement: Supplementary file 1 [file S0950268820000084sup001.docx]

# Epidemiology and Infection

**Q fever seroprevalence in Australia suggests one in twenty people have been exposed**

**H. F. Gidding, C. Q. Peng, S. Graves, P. D. Massey, C. Nguyen,
J. Stenos, H. E. Quinn, P. B. McIntyre, D. N. Durrheim, N. Wood**

**Supplementary Material**

**Supplementary Table S1.** **Numbers tested and crude Q fever seroprevalence by Australian state and territory**

| **State/Territory** | **N tested** | **N positive** | **% positive (95%CI)** |
| --- | --- | --- | --- |
| Australian Capital Territory | 31 | 4 | 12.9 (1.1-24.7) |
| New South Wales | 576 | 32 | 5.6 (3.7-7.3) |
| Northern Territory | 19 | 2 | 10.5 (0-24.3) |
| Queensland | 364 | 11 | 3.0 (1.3-4.8) |
| South Australia | 139 | 6 | 4.3 (0.9-7.7) |
| Tasmania | 10 | 6 | 20.0 (0-44.5) |
| Victoria | 441 | 32 | 7.3 (4.8-9.7) |
| Western Australia | 205 | 10 | 4.9 (1.9-7.8) |

**Supplementary Table S2.** **Numbers tested and crude Q fever seroprevalence by remoteness^a^**

| **Remoteness category^b^** | **N tested** | **N positive** | **% positive (95%CI)** |
| --- | --- | --- | --- |
| Very remote | 19 | 0 | 0 |
| Remote | 32 | 5 | 15.6 (3.0-28.2) |
| Outer regional | 204 | 11 | 5.4 (2.3-8.5) |
| Inner regional | 295 | 18 | 6.1 (3.4-8.8) |
| Major cities | 1233 | 65 | 5.3 (4.1-6.6) |

^a^ Remoteness Areas of Australia based on mapping postcode of residence to the Accessibility/Remoteness Index of Australia (ARIA) [1]

^b^ Two sera were excluded as they were missing postcode of residence.

**References**

[1] Hugo Centre for Migration and Population Research. The Accessibility/Remoteness Index of Australia 2011. Available at https://www.adelaide.edu.au/hugo-centre/services/aria. Accessed February 14 2019.)
